# Postharvest physiology and biochemistry of Valencia orange after coatings with chitosan nanoparticles as edible for green mold protection under room storage conditions

**DOI:** 10.3389/fpls.2022.1034535

**Published:** 2022-11-17

**Authors:** Khalid S. Alshallash, Mohamed Sharaf, Hosny F. Abdel-Aziz, Muhammad Arif, Ashraf E. Hamdy, Sobhy M. Khalifa, Mohamed F. Hassan, Mostafa M. Abou ghazala, Ahmed Bondok, Mariam T. S. Ibrahim, Khadiga Alharbi, Amr Elkelish

**Affiliations:** ^1^ College of Science and Humanities - Huraymila, Imam Mohammed Ibn Saud Islamic University (IMSIU), Riyadh, Saudi Arabia; ^2^ Department of Biochemistry, Faculty of Agriculture, AL-Azhar University, Nasr City, Cairo, Egypt; ^3^ Department of Biochemistry and Molecular Biology, College of Marine Life Sciences, Ocean University of China, Qingdao, China; ^4^ Department of Horticulture, Faculty of Agriculture, Al-Azhar University, Cairo, Egypt; ^5^ Department of Agriculture Botany, Faculty of Agriculture, Al‐Azhar University, Nasr City, Cairo, Egypt; ^6^ Department of Plant Pathology, Faculty of Agriculture, Ain Shams University, Cairo, Egypt; ^7^ Department of Biochemistry, Faculty of Agriculture, Ain Shams University, Cairo, Egypt; ^8^ Department of Biology, College of science, Princess Nourah bint Abdulrahman University, Riyadh, Saudi Arabia; ^9^ Botany Department, Faculty of Science, Suez Canal University Ismailia, Ismailia, Egypt

**Keywords:** citrus, nanomaterials, nano-chitosan, edible coating, storage period, fruit quality

## Abstract

Because of their unique features, nanomaterials have been proposed and have gained acceptance in postharvest applications in fruit. Increasing the storage life and improving the quality of Valencia oranges was investigated using nano-chitosan. A chitosan nanoparticle was prepared by using high-energy ball milling. Chitosan nanoparticles were characterized by Dynamic light scattering, FTIR spectroscopy and Surface morphology by transmission electron microscopy. Fully mature Valencia oranges were harvested and then coated with one of these concentrations (0.2, 0.4, and 0.8% nano-chitosan) and control. The fruits were stored under room storage conditions for 75 days. The quality parameters (fruit weight losses, fruit decay percentage, fruit firmness, total acidity, total soluble solids percentage and T.S.S./acid ratio, ascorbic acid content) were taken in biweekly intervals after 0, 15, 30, 45, 60, and 75 days. Beside the in vitro testing of antifungal activity of chitosan nanoparticles. According to the findings of the two succeeding seasons, the nano-chitosan 0.8% treatment showed the best effects and had the lowest rate of fruit weight loss, fruit deterioration, and T.S.S./acid ratio in comparison to the other treatments in both seasons. Furthermore, the 0.8% nano-chitosan reveled the highest levels of fruit hardness and fruit pulp firmness. Fruit weight loss, fruit deterioration, TSS, and TSS/acid ratio, as well as other metrics, were steadily elevated prior to the storage time. The best results were obtained when Valencia oranges fruits were treated with 0.8% nano-chitosan for 75 days at room temperature.

## 1 Introduction

Citrus is one of the most important fruit crops in Egypt and worldwide. Likewise, it ranks first in Egypt in terms of area and production (Abd-Elgawad, 2020)., which is the total cultivated area reached about 518920.84 acres harvested area is 308202.18 acres with exported quantity attained 1871150 tons (FAO, 2020). Citrus fruits are hailed as the best and most promising export fruits, as well as the most popular fruits in Egypt, due to their excellent taste, flavor and high vitamin C content (Ahmad et al., 2014). Citrus fruits are a major healthy contribution to the human diet due to their high nutritional value (Liu et al., 2012), especially vitamin C, which is critically essential for human fitness due to its increased resistance against influenza and decreased calcium oxalate accumulation in the kidneys (Haleblian et al., 2008).

The Valencia orange [*Citrus sinensis* L. (Osbeck)] is the most exported citrus fruit among other citrus species in Egypt (Sameh et al., 2014), and its fruit products are largely consumed as juice and are in demand worldwide (FAO, 2020). The late cultivar Valencia orange under Egyptian conditions is the dominant cultivar grown on newly reclaimed desert lands (Mansey et al., 2021). Late harvest leads to deterioration in the quality of the fruits due to heat stress (Zhang et al., 2022). Therefore, storage is critical to maintain the fruits’ shape and quality and to reduce some fungal infections (Mariod et al., 2018). Using safe materials may be a solution for maintaining the quality of the fruit (Ramos et al., 2013). Moreover, eco-friendly and sustainable approach could be a potential method to reduce the damage caused by fungicides and may be economically advantageous when used in disease management programme (Zahid et al., 2015).

Nanoparticles for chemicals attract scientific interest because they effectively bond amid bulk materials and atomic or molecular constructions (Roberto et al., 2019). A bulk substantial should have persistent physical characteristics unrelated to its size, but at the nanoscale, a change in size-dependent characteristics is often noted. In addition, the percentage of atoms current on the superficial becomes significant compared to the number of atoms in the mass of the material (Noordeen et al., 2013). Nanosized materials are employed because of their large surface areas, which result in strong reactivity, a powerful catalyst for plant metabolism, improved cell penetration, and increased plant activity (Raliya et al., 2014). Consumer demands are increasing daily for high-quality and microbiologically safe food and a longer product shelf life. These goals challenged scientists to develop new food preservative strategies. One of these proposals is to employ edible film made from polysaccharides and lipids as edible covering materials (Olivas and Barbosa-Cánovas, 2008; Raliya et al., 2014).

Chitosan is an ordinary carbohydrate polymer derivative by deacetylation of chitin [poly-β-(1→4)-Nacetyl-D-glucosamine], which is the main component in crustacean shells such as crab, shrimp, and crawfish. In addition, In Egypt, chitosan is the recycled material and its cost is low (Szlachta et al., 2020). Three related functional groups can be found in chitosan at the C-2, C-3, and C-6 positions: amino groups, primary hydroxyl groups, and secondary hydroxyl groups. Aranaz et al. (2012), considered the physic-chemical behavior and functional chattels of chitin and chitosan and planned specific presentations in drug supply, tissue culture, functional food, food protection, biocatalyst, immobilisation, wastewater treatment, molecular printing, and metallic-nano-composites. A thin layer of biopolymer containing chitosan was used as an antimicrobial agent (Aranaz et al., 2012; Szlachta et al., 2020), possessing film-forming qualities and antifungal activity (Dainelli et al., 2008). It is a high molecular weight cationic polysaccharide (Arvanitoyannis, 2008).

Films made with chitosan offer high mechanical characteristics and gas selective permeability (CO_2_ and O_2_); however, its limited use is due to its high water vapour permeability (Campos et al., 2011). Chitosan is the most widely used polysaccharide as a basic film-forming and coating material in food packaging because of its inherent edible and antibacterial qualities; therefore, it has been widely used to protect and increase fresh foods’ shelf life and quality (Campos et al., 2011). Ali et al. (2014) showed that 1.0% chitosan concentration with 600 nm SCD has the potential to be used as a bio fungicide against anthracnose of dragon fruit plants.

In this context, covering strawberry fruits with edible chitosan has shown to be a secure and reliable way for extending their shelf life (Vargas et al., 2006). In addition, it maintains the overall post-harvest quality of tomatoes where, they show that quality was generally maintained during the storage period because, in contrast to the controls, changes in the major physical and biochemical quality parameters took longer to occur (Mustafa et al., 2013). Moreover, chitosan resulted in better control of post-harvest rot in apple, that coating with chitosan could be practical and useful for prolonging the chemical and microbial shelf lives of fresh-cut apples during refrigerated storage (Özdemir and Gökmen, 2019) . In addition, an edible coating of oranges and lemons *C. saitoana* with 0.2% glycolchitosan reduced green mould incidence equally compared with those of control and other treatments (El-Ghaouth et al., 2000).. Also, the quality properties of lemon fruit samples were improved by using a coating of chitosan-clay nano-composite during storage (Taghinezhad and Ebadollahi, 2017). Moreover, The edible chitosan nanoparticle coatings delayed the ripening of the grapes, which decreased weight loss, soluble solids, and sugar contents. It also boosted moisture retention and preserved the treatable acidity values and sensory qualities (Castelo Branco Melo et al., 2018). Furthermore, studies of the physical properties of ripening bananas revealed that fruits coated with chitosan nanoparticles 0.2% experience a 2-3-day delay in skin discoloration compared to controls (Esyanti et al., 2019). In addition, when compared to control and other treatments, pomegranate fruits treated with chitosan (CH) at 1% and CH 2% showed the lowest decay incidence and weight loss (Molaei et al., 2021) and (Hasheminejad and Khodaiyan, 2020). Additionally, the addition of chitosan (CTS) at 1% (w/v) or glycine betaine-coated chitosan nanoparticles (CTS-GB NPs) at 0.5 and 1% (w/v) reduces chilling injury, maintains nutritional quality, and increases the fruit’s storage ability and shelf life (Mahmoudi et al., 2022). Chitosan 1% treatment improved the antioxidant capacity, hardness, and total soluble solids of persimmon fruits while delaying the deleterious effects of chilling stress (Nasr et al., 2021). Zahid et al. (2019) investigated that 1.0% chitosan with 600 nm droplet size showed better results in terms of reduction in diseases severity on dragon fruit plants.

*Penicillium digitatum* Sacc. is one of the most destructive diseases in citrus fruits, causing almost 90% of all production losses at post-harvest treatment. Despite the use of fungicides and the expansion of biological control strategies (El-Ghaouth et al., 2000) green mould infection pressure on storage citrus products continues to be strong. *P. digitatum* causes green mould, the world’s most common post-harvest disease, damaging citrus production in the packing house, during transportation, and at the market (Schmidt et al., 2010). The function of chitosan appears to be a direct antifungal effect on fungi, consequently increasing their susceptibility to the host chitinase and β-1,3-glucanase as well as lytic enzymes in papaya fruits (Ali et al., 2012). Harvested fruits are normally preserved for fresh consumption before reaching the market, and fungal disease infection is the major cause of fresh citrus fruit decline throughout the post-harvest period (Ballester et al., 2010). Chitosan, chitin-derived, has long been recognized as a natural antibacterial that may kill many bacteria, fungi, and yeasts. Furthermore, chitosan has been shown to stimulate various host defensive responses, providing defense against infection in various host plants (Romanazzi et al., 2012). Maqbool et al. (2010) reported that 10% (w/v) GA + 0.75% (w/v) Chitosan may be particularly appropriate for banana growers and exporters as a post-harvest pesticide treatmentTo improve chitosan’s performance, an ionic gelation technique was performed to minimise the particle size entering the nanometer range However, limited studies are available regarding the application of Valencia orange fruits with chitosan nanoparticles. Hence, the present study aims to elucidate the antifungal effect of the chitosan nanoparticles *in vitro* versus *P. digitatum*. Furthermore, Testing the protective effect of the chitosan nanoparticles coating as a safe material on storage life, quality, and the reduction of some fungal infections of Valencia oranges. This can be demonstrated by measuring quality parameters (fruit weight losses, fruit decay percentage, fruit firmness, total acidity, total soluble solids percentage and T.S.S./acid ratio, ascorbic acid content).

## 2 Materials and methods

### 2.1 Sample collections

In this experiment, Valencia oranges (*Citrus sinensis* L. (Osbeck) trees were studied throughout the 2020 and 2021 growing seasons. The post-harvest laboratory of the Horticulture Department, Faculty of Agriculture in Cairo, and Al-Azhar University, Egypt, hosted the experiment. A private orchard in Wadi Almollak, Ismailia Governorate, Egypt (30°35’N, 32°14’E, altitude 13m above sea level), provided the fruits. The selected trees in age, budded on Volkamer lemon (C. *volkameriana* Ten. and Pasq.) rootstock, cultivated in sandy soil with drip irrigation, spaced 3 to 5 meters apart (280 trees/feddan), and all were 10 years old.

### 2.2 Sample preparation

Ninety fruits for each treatment were picked when they were fully mature when peel color at maturity ranges from light to deep orange according to Wardowski et al. (Wardowski, 1995), put in cartons, and brought straight to the lab, through an hour from harvesting the fruits from orchard to the beginning of the treatment in lab. The selected fruits were sound and devoid of pathological and physiological conditions. After being cleaned with tap water, the fruits were immerged in Borax solution at concentration 1% for 5min for disinfected, the fruit was air-dried before being treated. The experiment had three repetitions and a factorial (44) completely randomized design. Each replication had 30 uniform fruits. Chitosan nanoparticles (CNPs) coating application is 0.2% (T1), 0.4% (T2), and 0.8% (T3), under room storage times, (0, 15, 30, 45, 60, and 75 days).

CNPs-treated fruits were air-dried while in the control treatment, and water was used instead of the CNPs. All treated and untreated fruits were packed in perforated (0.06% of the area) 20 micron-thick, low-density polyethene (LDPE) bags and stored at room conditions for 75 days. The quality parameters were recorded and analyzed biweekly after (0, 15, 30, 45, 60, and 75 days).

### 2.3 Chitosan nanoparticles preparation

CNPs were synthesized by the ionic gelation technique. To make the CNPs, a chitosan (C) acidic solution was mixed with a basic tripolyphosphate (TPP) solution at room temperature (18 ± 2 °C.) Chitosan solutions were prepared by dissolving 1 g of chitosan in 100 mL of 1.0% aqueous acetic acid and stirring the liquid until it turned transparent. After that, NaOH was employed to reduce the pH to 5.5 (0.01 N). The sodium tripolyphosphate solution (1.0%) was then added dropwise to the chitosan solution while stirring. The TPP-initiated ionic gelation technique was employed to commence the spontaneous synthesis of CNPs. The final suspension was swirled for 30 minutes at room temperature (Arif et al., 2021)

### 2.4 Distributions and characterizations of chitosan NPs

#### 2.4.1 Dynamic light scattering measurement

The mean of particle size and z potential nanoparticles for the formulations were measured by Dynamic light scattering (DLS) using (Malvern Instruments, UK). For size estimation, 3 ml of bare CNPs was diluted in deionized water and placed in a cell cuvette and scanned four times to get an average reading. The mean ± SD was obtained after three measurements (Sharaf et al., 2021).

#### 2.4.2 FTIR spectroscopy

FT-IR spectrometer (JASCO FT-IR 4100 spectrometer, Hachioji, Tokyo, Japan) was used to examine the functional groups contained in prepared samples. Potassium bromide (KBr) mixed with prepared samples. A disc was loaded at high pressure and measured at a wavelength of 400–4000 cm−1 with a resolution of 4.0 cm−1 (Sharaf et al., 2022).

#### 2.4.3 Surface morphology

CNPs were examined using transmission electron microscopy (TEM) technology (TEM.; TOPCON002B; Tokyo, Japan). By simply placing a little amount of the sample on the grid and wiping away any extra solution using blotting paper, thin films of silver nanoparticles were produced on a copper grid covered with carbon (Sharaf et al., 2022).

### 2.5 Data recorded

All treated fruits of Valencia orange were stored at room conditions (18 ± 2 °C and 55%, R.H.) directly after harvest (day 0) and evaluated at biweekly intervals. The decay incidence of each treatment was monitored after 15 days during each season.

#### 2.5.1 Fruit weight losses percentage (F.W.L. %)

The fruits were weighed both before and after each time of room storage to determine the original weight. The formula for calculating F.W.L. % is as follows:


FWL %=(Wi –Ws)/Wi) X 100

Where, Wi = fruit weight at the initial date, and Ws = fruit weight at the sampling date according to Hazali et al. (2013).

#### 2.5.2 Fruit decay percentage (F.D. %)

The percentage of disordered fruits, including all spoiled fruits resulting from rots, fungus, bacteria and pathogens, were assessed. The defects were determined by calculating the number of decayed fruits at the harvesting date expressed as a percentage of the initial fruit number according to El-Nagar et al. (Elnagar et al., 2021).

#### 2.5.3 Fruit firmness (FF lb./inch^2^)

According to Ibrahim and Gad, F.F. was determined in peel and pulp using a pressure tester (Digital force-Gouge Model FGV-0.5A to FGV-100A. Shimpo instruments) (Ibrahim and Gad, 2015).

#### 2.5.4 Total acidity (T.A.) (%)

T.A. was determined using titration and expressed as citric acid according to Hazali et al. (2013).

#### 2.5.5 Total soluble solids percentage (T.S.S. %) and T.S.S./acid ratio

For total soluble solids percentage determination the juice of fruit were prepared by a juicer according to Nasr (Nasr et al., 2021) therefore, TSS was measured (in triplicate) using a Digital refractometer (ATago. PAL-1. Japan) according to AOAC (McKie and McCleary, 2016). T.S.S./acid ratio was recorded by dividing the T.S.S. value by the total acidity value according to Ibrahim and Gad (2015).

#### 2.5.6 Ascorbic acid content (vitamin C) mg/100 ml juice

It was discovered using the AOAC-recommended substrate of 2, 6 dichlorophenolendophenol with a 2% solution of oxalic acid (AOAC Association of Official Analytical Chemist, 2016).

### 2.6 Antifungal properties

#### 2.6.1 Pathogen culture

*P. digitatum* was isolated from rotted infected citrus fruit with green spores on it and cultured on media of potato dextrose agar (P.D.A.). After 10 days: Spores were collected from old plates, washed with sterile distilled water using a swab and filtrated to remove most of the debris and hyphal fragments. The concentration of spores suspension was adjusted by dd water containing Tween 80 (0.05% w/v) to 106 mL before inoculation and counted using the hemacytometer (Macarisin et al., 2007).

#### 2.6.2 Antifungal activity of CNPs *in vitro*

The antifungal effects of chitosan versus *P. digitatum* were assessed using P.D.A. plates with three concentrations of chitosan. 50 µl of spores suspension (106) was spread on the surface of plates then 20 µl of T1, T2, and T3 of chitosan was tested on the disk of filter paper in the middle of plates. The results were observed after 3 days when the control plates were grown well, and the growth inhibition zone was calculated by cm.

#### 2.6.3 Antifungal activity of CNPs *in vivo* on fruit inoculation and disease assessment

Fruits were wounded around the end of blossom in two different sites on oranges by making piercing or holes depth of 5 mm with a 1.25 mm diameter needle. 50 µl of spores suspension of *P. digitatum* or Tween 80 solution (0.05% w/v) were pipetted into a-holes for treatment and control, and then fruits were kept at room temperature before disease evaluation. For CNPs treatment: three concentrations of chitosan (0.2%, 0.4%, and 0.8%) were coated on the fruits before and after treatment by the spores suspension of *P. digitatum*. Diseases assessment was evolution when the control was full infected. Each treatment in the experiment was performed in three replicates of 5 fruits, and the results were measured as the percentage of inhibition disease (El Guilli et al., 2016).

### 2.7 Statistical analysis

The obtained data were analyzed using Co-stat 3.4 software with tow-way analysis were the first factor revers to the concentration of Nano chitosan and the second factor was storage times, as a completely randomized design. According to Snedecor and Cochran (1990), the differences among treatments for all tested parameters were compared with Duncan’s multiple range tests at a 5% level.

## 3 Results

### 3.1 CNPs distribution and characterization

#### 3.1.1 DLS

**Figure 1:**
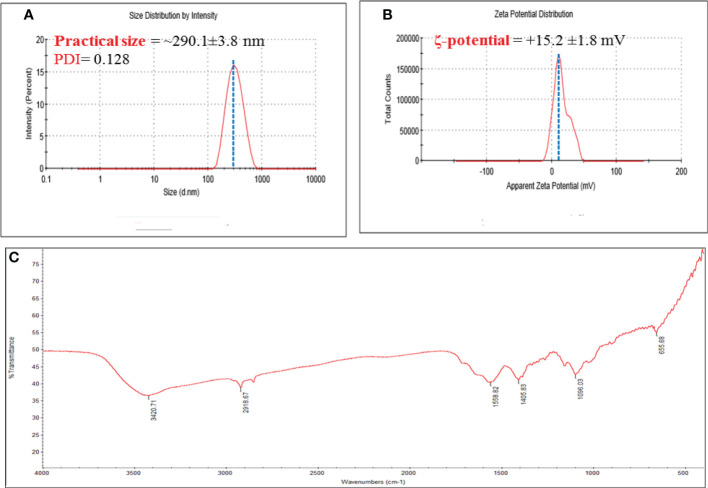
**(A)** Hydrodynamic size, and polydispersity index (PDI); and **(B)** ζ-potential of chitosan NPS. Numerical data is reported as mean ± SD ζ-potential (n=3) and particle size and PDI (n=4); and **(C)** FT-IR spectra of Chitosan NPs extract.

DLS was used to measure PS, PDI, and ζ-potential of bare CNPs. The mean values recorded for all the systems showed a PS distribution in nanometers as shown in **Figure 1**. The size of CNPs was about ~290.1 ± 3.8 nm, while the PDI were 0.128 (**Figure 1A**).

Furthermore, a very narrowly distributed particle possesses PDI values of about 0.01 - 0.3 which is ideal for stability and uniformity of dispersion (Park et al., 2016). The stability of nanoparticles is generally predicted by their ζ-potential values; here, the determined ζ-potential values were +15.2 ± 1.6 mV for chitosan NPs (**Figure 1B**).

Additionally, the ζ-potential value that is higher than<10 mV is considered to be stable due to electrostatic balance. The higher of the positive ζ-potential charge of chitosan NPs could be attributed to the ionization of the amino functional groups (-NH2) in the capping moieties at acidity pH (AbdelHamid et al., 2013), the high positively charge forming a repulsive barrier that helped to avoid the aggregation and improve the colloidal stability of chitosan NPs.

#### 3.1.2 FTIR analysis

FTIR spectroscopy was used to investigate the functional groups of CNPs, as depicted in **Figure 1C**. The O-H group of stretching vibrations caused a peak at 3420 cm1 for the primary functional group of chitosan. The existence of absorption peaks at 1559 and 1405 cm1 is attributed to protonate the amino (NH2) group N-H bending vibration and the alkyl group C-H bending vibration. The absorption peaks at 1096 and 655 cm1 are attributed to the glucopyranose ring in the chitosan matrix due to the anti-symmetric stretching vibration of C-O-C bridges. Oh et al., 2019 observed similar peaks of produced CNPs against tomato phytopathogens (Oh et al., 2019).

#### 3.1.3 Surface morphology of chitosan nanoparticles by TEM analysis

**Figure 2:**
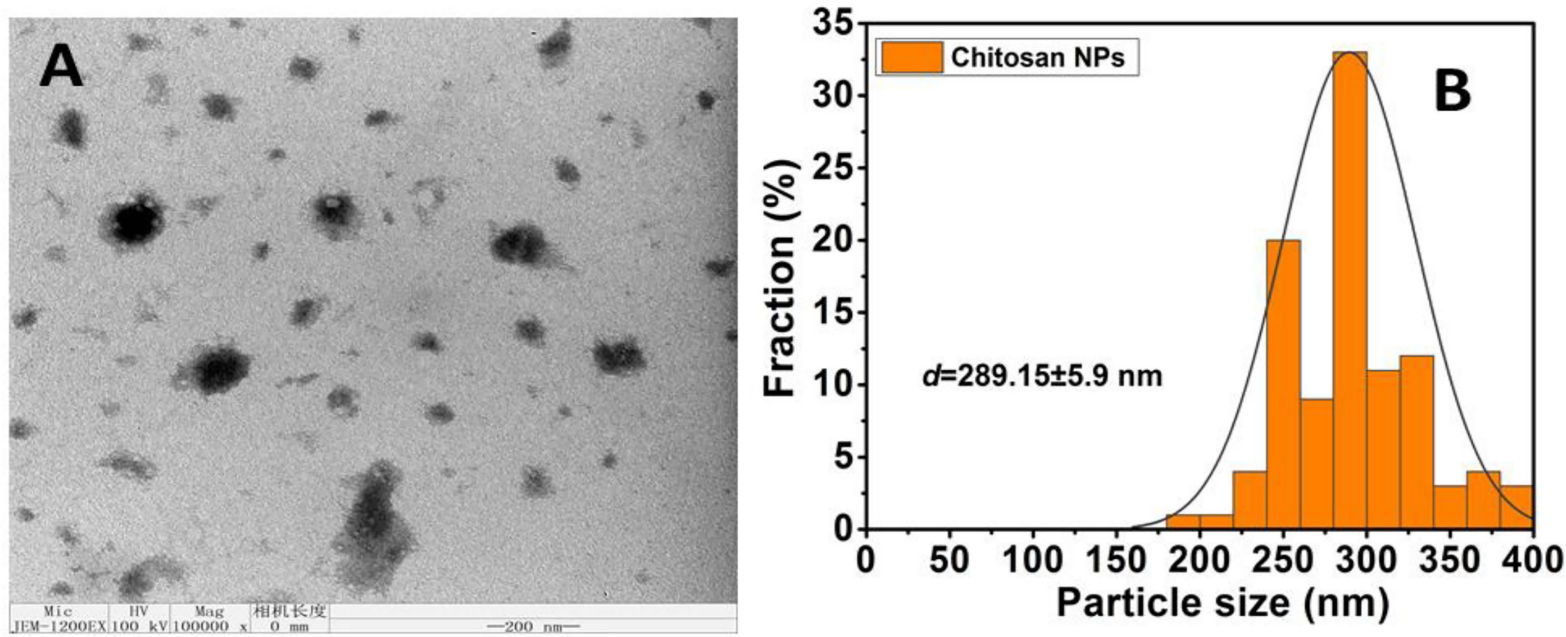
**(A)** Average particle size and size distribution of prepared samples Chitosan NPS measured by TEM; (scale bar =200nm); **(B)** Data of size distribution is presented as means ± SD (n = 3).

TEM analysis was used to further investigate the surface morphological structure. The TEM pictures of CNPs are illustrated in **Figure 2** at a reduced magnification scale of 200 nm. CNPs morphological structure demonstrated a large particle size, spherical-like structure, and particles in an agglomerated condition with non-aggregation, and this finding was compared to those obtained using DLS. Furthermore, the size homogeneity of the nanoparticles can be observed (**Figure 2A**). The diameter mean of size distributions are presented in **Figure 2B**.

### 3.2 Effect of chitosan NPs on the physical properties of fruit under room storage conditions

**Figure 3:**
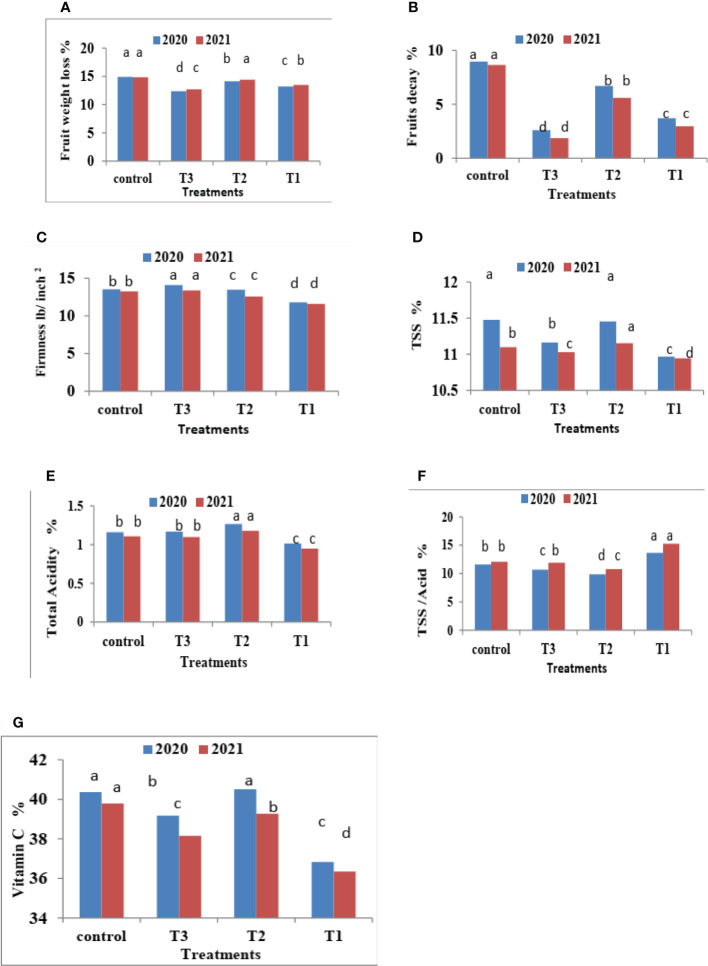
Effect of CNPs treatment on quality of Valencia oranges. **(A)** FWL, **(B)** % FD%, **(C)** FF%, **(D)** TSS %, **(E)** TA %, **(F)** TSS/acid ratio and **(G)** VC% in 2020 and 2021 seasons. Control: without Chitosan nanoparticles (CNPs) coating, 0.2% (T1), 0.4% (T2), and 0.8% (T3) Chitosan nanoparticles (CNPs) coating, Bars with different letters are significantly different according to DMRTs at 0.05 level.

**Figure 4:**
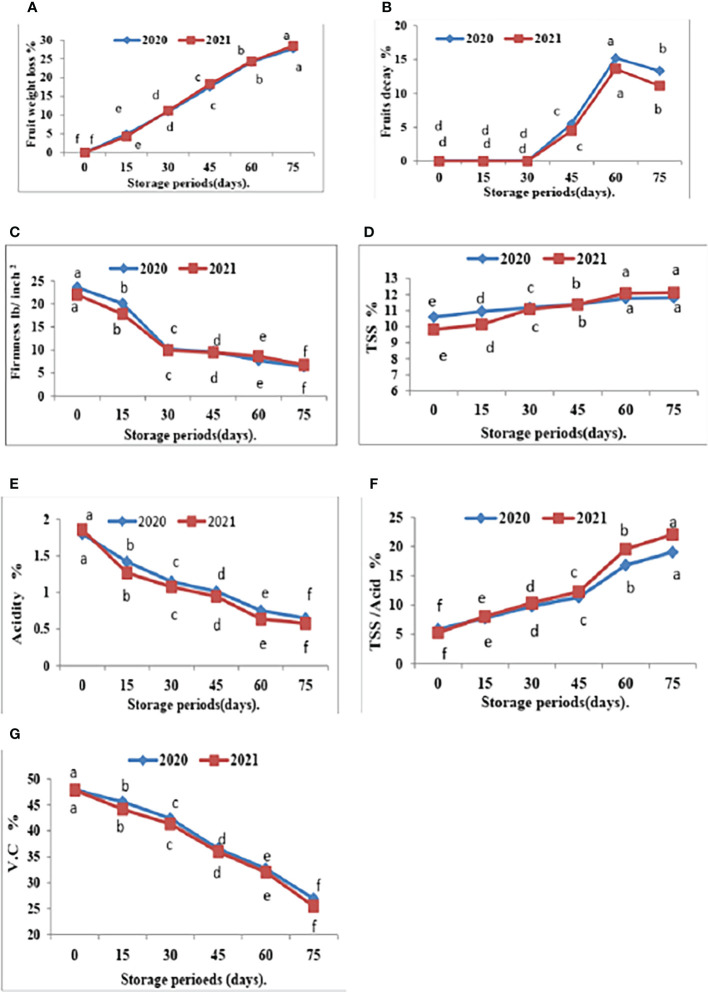
Effect of room storage period on Valencia oranges quality after treatment with CNPs. **(A)** FWL % **(B)** FD %, **(C)** FF%, **(D)** TSS %, **(E)** TA %, **(F)** TSS/acid ratio and **(G)** VC in 2020 and 2021 seasons Control: without Chitosan nanoparticles (CNPs) coating, 0.2% (T1), 0.4% (T2), and 0.8% (T3) Chitosan nanoparticles (CNPs) coating. Points with different letters are significantly different according to DMRTs at 0.05 level.

**Figure 5:**
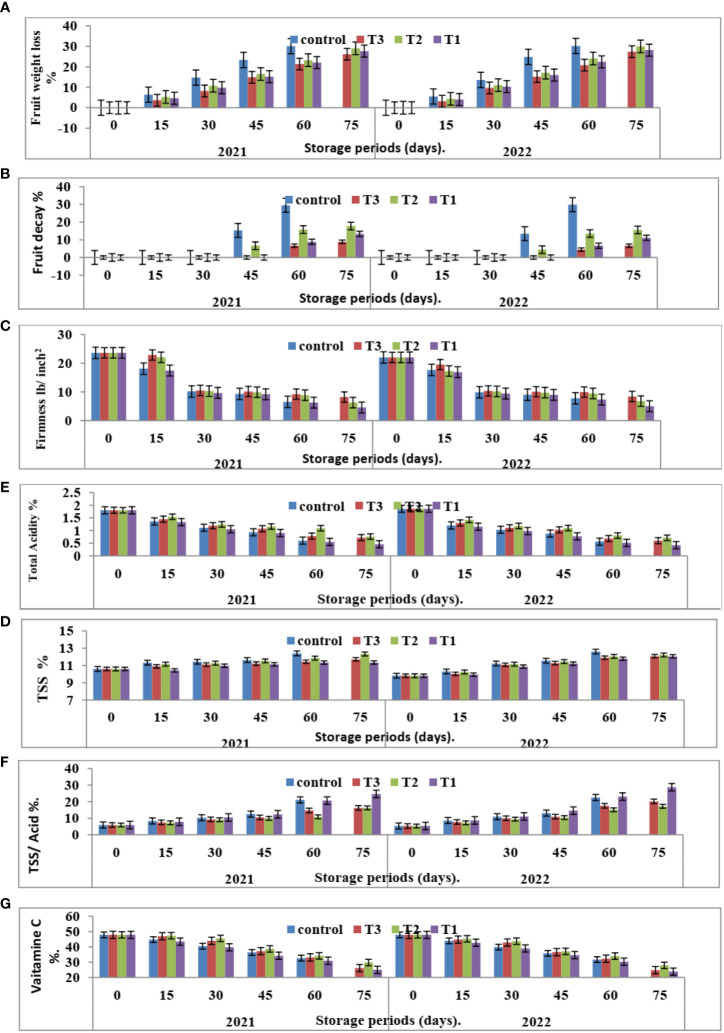
Effect of the interaction between nano CNPs concentrations and room storage period on Valencia oranges quality. **(A)** FWL % **(B)** FD %, **(C)** FF%, **(D)** TSS %, **(E)** TA %, **(F)** TSS/acid ratio and **(G)** VC in 2020 and 2021 seasons. Control: without Chitosan nanoparticles (CNPs) coating, 0.2% (T1), 0.4% (T2), and 0.8% (T3) Chitosan nanoparticles (CNPs) coating. Bars with different letters are significantly different according to DMRTs at 0.05 level.

It is important to note that the storage period of Valencia oranges treated with CNPs under room conditions (18 ± 2°C and 55%, R.H.) extends to 75 days in both seasons studied in 2020 and 2021. The results are shown in **Figures 3**–**5**.

#### 3.2.1 Fruit weight loss % and decay %

**Figure 3** indicates that CNPs treatment significantly decreased the percentage of FWL% and FD% in Valencia oranges compared to the control treatment. Whereas CNPs treatment at a concentration of 0.8% gave the lowest value (12.37 and 12.70) in FWL % (**Figure 3A**) and FD% (**Figure 3B**) compared to the other treatments.

Furthermore, with respect to the effect of the storage period, data in **Figures 4**, **5** showed that Valencia orange fruit FWL% (**Figure 4A**) and FD% (**Figure 4B**) were increased by increasing the storage period under room conditions, where fruits stored for 75 days recorded the highest values compared to those stored for 15 days which gained the lowest values of FWL% and FD % (**Figures 5A, B**).

After 75 days of storage, CNPs treatment at a concentration of 0.8% recorded the lowest significant percentage of fruit FWL% and FD% compared to the control, which had the highest FWL% and FD% in both seasons. The reducing value of FWL% in Valencia oranges at different storage periods was related to fruit post-harvest treatments by CNPs compared to the control in both seasons. From these results, the decreasing fruit FWL% and FD% may be attributed to controlling decay and its damages through chitosan against post-harvest diseases, making a thin film surrounding the fruit peel, and inducing a modification of the atmosphere around the fruits (Author et al., 2017).

#### 3.2.2 Fruit firmness (lb./inch^2^)

It is clear from **Figure 3C** that CNPs treatments significantly maintained the quality of Valencia oranges. Higher concentrations of CNPs maintained FF, whereas the lowest values of FF were recorded by CNPs 0.2% treatment compared to CNPs 0.8% treatment which gave the highest values of FF followed by the control treatment in both studied seasons.

Furthermore, FF gradually decreases with an increased storage period. The highest FF values were obtained after 15 days of room storage and then values gradually decreased.

The interaction between CNPs concentrations and the storage period on fruit quality properties in **Figure 5C** showed that the highest CNPs concentration of 0.8% gave high FF in 15 days then decreased as compared to CNPs 0.4% in both seasons. Storing fruits for 75 days significantly gave the lowest FF in all treatments, whereas the highest FF was shown in the CNPs (0.8% treatment compared to all treatments in the two seasons.

#### 3.2.3 The percentages of total soluble solids (TSS %), total soluble solids (TSS/acid ratio), total acidity (TA %), and ascorbic acid (VC %)

Data in **Figure 3** indicates that CNPs treatments significantly maintained the quality of Valencia orange fruits. Higher concentrations of CNPs (0.4%) increased TSS % (**Figure 3D**) and TSS/acid ratio (**Figure 3E**) compared to the other treatments. On the contrary, the treatment with CNPs of 0.2% gave the lowest values of TSS % and TSS/acid ratio in both seasons. The lowest concentrations of CNPs (0.2%) decreased the percentages of TA (**Figure 3F**), and VC (**Figure 3G**), compared to the other treatments.

The comparisons of the effect of room storage for different periods (0, 15, 30, 45, 60, and 75 days) are shown in **Figure 4**. TSS % (**Figure 4D**) and TSS/acid ratio (**Figure 4E**) gradually increase with an increased storage period; the highest values were recorded in fruits stored for 75 days. On the contrary, the treatment with CNPs of 0.4% gave the highest value percentages of TA% (**Figure 4F**) and VC (**Figure 4G**) in both seasons. The TA and VC gradually decreased with an increased storage period, and the lowest values were recorded in fruits stored for 75 days. Compared to other nutrients, ascorbic acid is a relatively sensitive nutrient quality component that declines quickly due to oxidation during storage (Jawad et al., 2013).

The interaction between nano-chitosan concentrations and storage period on fruit quality properties in **Figure 5** showed that the highest CNPs concentration of 0.4% gave high TSS (**Figure 5D**) and VC (**Figure 5G**) in 75 days. On the other hand, the lowest CNPs of 0.2% gave the lowest TA% (**Figure 5F**). At the same time, nano-chitosan treatment of 0.2% gave the highest value of TSS/acid ratio (**Figure 5E**) and TSS (**Figure 5D**) at 75 days compared to all treatments in the two seasons of 2020 and 2021.

##### 3.2.3.1 *In Vitro* antifungal activity assay

**Figure 6:**
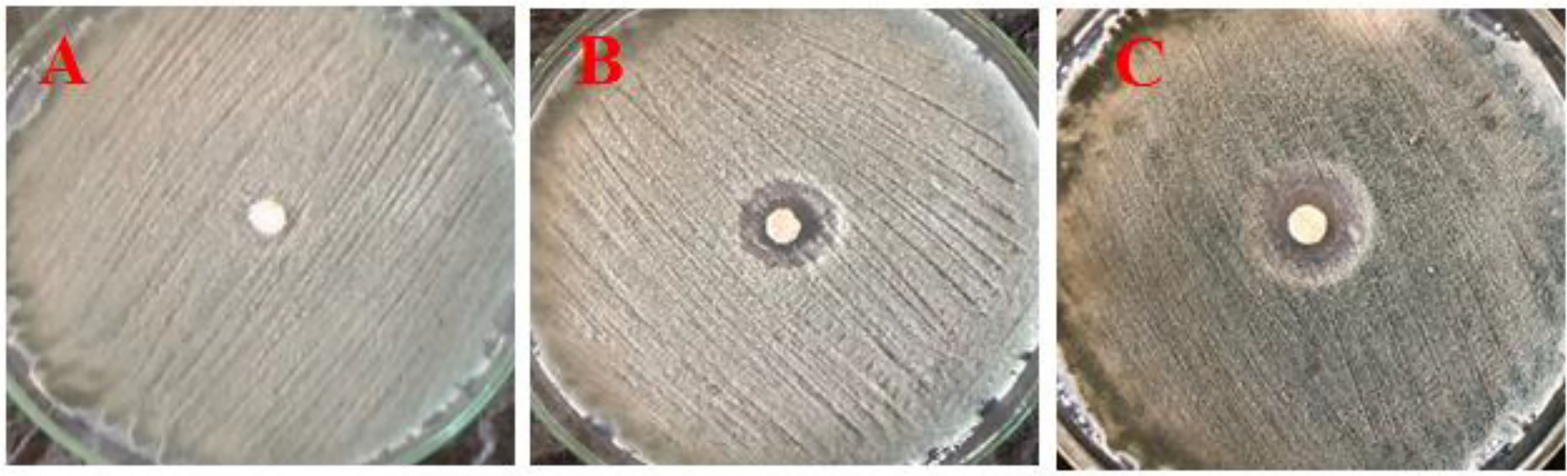
Effect of different concentrations of nano CNPs (invitro) **(A)** T1 with 0.2%, **(B)** T2 with 0.4%, and **(C)** T3 with 0.8% of CNPs on mycelia growth of *P. digitatum in vitro* (bar = 1 mm).

*In vitro*, antifungal results showed that the inhibition zone of *P. digitatum* growth was increased when the concentration of CNPs increased, and the inhibition zone was observed in PDA plates (**Figure 6**). Also, the inhibition zone values of CNPs concentrations were measured and recorded. Thus, a 0.4% concentration was considered the lowest concentration with an effect without a clear zone (12 mm), whereas 0.8% had a clear zone (19 mm). As the concentration of chitosan increased, fungal growth was reduced.

##### 3.2.3.2 *In Vivo* disease assessment on fruits

**Figure 7:**
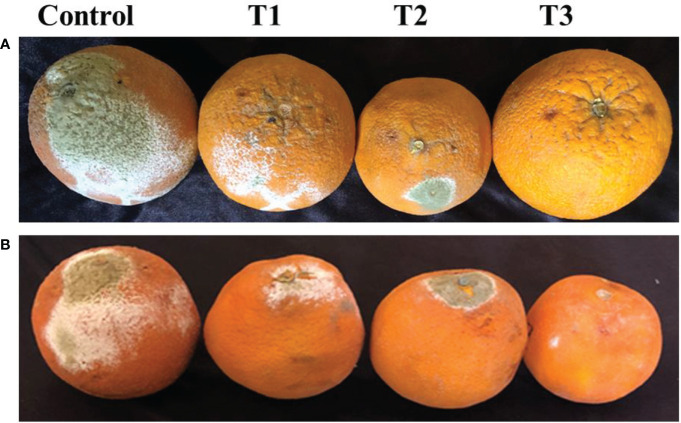
*In vivo* phytopathogenicity of *P. digitatum* on Citrus fruits. **(A)** after and **(B)** before being infected with various concentrations of treatment. A) T1 with 0.2%, **(B)** T2 with 0.4%, and **(C)** T3 with 0.8% of CNPs the increase in CNPs concentration significantly affected the fungus growth on fruit surfaces (bar = 1 cm).

The effects of three concentrations of CNPs were observed before and after being infected by *P. digitatum*. “*In vivo* assays indicated that when uncoated fruits were inoculated with the pathogen *P. digitatum* spores, the fruits showed rapidly penetrated puncture wounds within four days. Then mycelia widely invaded extensively healthy tissue around the injured areas in the control, and the sporulation can be seen as abundant on the fruits which were covered completely by white mycelium followed by green spores (**Figure 7**). However, growth of *P. digitatum* was greatly inhibited in CNPs -coated fruit based on chitosan concentration, with a decrease of the colonized area by 95 percent at 0.4%; after that, the fungus development was prevented beginning from 0.8% which completely starting halted the fungus growth was. Also, the treatments of CNPs -coated fruit after (**Figure 7A**) being infected by *P. digitatum* were better than before (**Figure 7B**).

## 4 Discussion

Chitosan’s effective film-forming ability is widely utilized to preserve, enhance the quality, and extend the shelf life of fresh foods (Kumar et al., 2021); chitosan coating can increase the storage period of fruits and vegetables because it forms a semi-permeable membrane on the surface of the fruit. This lowers transpiration and water loss, and delays fruit ripening (Wardowski, 1995; Maqbool et al., 2010) as well as modulating the gas exchange between the external environment and internal gas formation.

CNPs with modified atmosphere packaging maintained fruit quality (Simões et al., 2009). Water loss may be one of the main causes of deterioration, as it not only results in direct losses in quantities but also causes loss of appearance due to wilting and shrivelling, and also loses nutritional quality (Kader).

CNPs has been shown to reduce water loss in a variety of horticultural products, including mangoes, bananas and peaches (AOAC Association of Official Analytical Chemist, 2016; El Guilli et al., 2016). Chitosan produces a coating film, which is known to frequently inhibit CO_2_ production and, as a result, the production of the commodity ethylene (Galus and Kadzińska, 2015). Chitosan’s combination of antifungal and eliciting properties is what gives it its inhibitory effect on decayed fruit. Chitosan can lessen the severity of disease, possibly by enhancing PAL and PPO activity, lignification brought on by increased biosynthesis of phenolic compounds or induced secondary metabolites, and SAR (Uthairatanakij et al., 2007). The loss of fruit firmness of the CNPs-treated fruits was delayed, signifying that Valencia orange fruit ripening was delayed, resulting in firmer fruit (Algarni et al., 2022). According to (Jin et al., 2018), the amount of insoluble protopectin degraded to simple pectin increased with the development of the storage period. The loss of firmness is associated with the degradation of the cell wall by the enzymes polygacturonasis and pectinatilesterasis plus water loss (Ren et al., 2020).

The results obtained by (AOAC Association of Official Analytical Chemist, 2016), on apples determined how fruit firmness decreases with increased storage time. Numerous examples show that peaches, apricots, papayas, and other fruits were delayed throughout the storage time, and other studies demonstrate that the treated fruit was firmer at the conclusion of storage (Vargas et al., 2006; AOAC Association of Official Analytical Chemist, 2016; Park et al., 2016).

The acidity and ascorbic acid were slowly reduced in the chitosan-treated fruits at the end of the storage period, associating this decrease with loss of eating quality (Jawad et al., 2013; Author et al., 2017). Decreased TA and V.C. during the storage period might be due to the destruction of organic acids through oxidation and their consumption in the respiration processes within fruit tissues. Progress of the storage period was found to raise the respiration rate of the fresh fruits (Kader and Ben-Yehoshua, 2000).

The effect of chitosan treatment on soluble solid contents was probably due to the slowing of respiration and metabolic activity, thus delaying the maturation process, modification of the endosphere by reducing O_2_ and raising CO_2_, and suppressing ethylene development (Conte et al., 2013). These results are consistent with (Kader; Zahid et al., 2015), who found that navel oranges treated with chitosan significantly increased T.S.S. during storage periods.

At the end of the storage period, several studies reported that TA increased over the chitosan-treated commodity (strawberries and peaches). At the same time, in other crops such as mangoes and longans, acidity gradually decreased, linking this decrease to a loss of eating quality (Vargas et al., 2006; Eshghi et al., 2014; El-Gioushy et al., 2022). After storage, the TSS and TSS/acid ratio dissolved solids ratio of chitosan-treated fruit differed by commodity. Lower TSS % on control fruit was reported in mangoes and bananas coated with chitosan, while higher values were reported on treated peaches. However, other studies recounted that the “TSS” of CNPs-dipped papayas was the same as the untreated fruit (Sciences, 2012; Mustafa et al., 2013). VC content was also evaluated in mangoes and peaches treated with chitosan (Eshghi et al., 2014; Galus and Kadzińska, 2015). In those studies, the VC content of mangoes decreased slowly during the storage period and was lower than in the unprocessed fruit. However, for Valencia oranges, the ascorbic acid content was higher in the chitosan-treated fruit compared to untreated (control), these results being similar to Romanazzi et al. (2017), who reported that the vitamin C content was higher than the untreated fruits coated with chitosan. CNPs coating benefits protracted food storage, especially for fruits and vegetables. According to previous studies, fruits including strawberries, peaches, pears, and litchi might be coated with CNPs to prevent and avoid post-harvest disease (El Guilli et al., 2016).

CNPs has an antifungal effect on host-pathogen interactions and the ability to induce plant immune defenses (Romanazzi et al., 2013). The studies indicate that the amount of inhibition growth is highly correlated with chitosan concentration, and the inhibition zone of *P. digitatum* on PDA plates increased as CNPs concentration increased. Several fungi were inhibited from growing *in vitro* when chitosan was used. Therefore, when CNPs concentration increased, *Penicillium*, *Aspergillus niger*, *Rhizopus stolonifer*, *Botrytis cinerea*, and *Sclerotinia sclerotiorum* showed reduced radial growth (Bautista-Baños et al., 2006; Yin et al., 2013). According to several studies, CNPs prevents post-harvest rot disease during storage, delay infection, and decreases growth. This study agrees with previous studies that chitosan coatings are effective against the green post-harvest in citrus fruits by showing a significant delay of fungal infection and decay when fruits were coated by chitosan (Long et al., 2018).

## 5 Conclusion

Valencia oranges are non- climacteric fruit with a short post-harvest storage life due to quality decline. Coating the Valencia oranges is one of the most important methods for preserving their quality. Chitosan nanoparticles were effective in fighting dangerous germs in tests. Nanoscale materials have evolved into cutting-edge antibacterial agents. Using CNPs to increase the shelf life of Valencia oranges while they are stored appears to be very promising in this sector of the food industry. According to a thorough comparison and assessment, Valencia oranges coated with CNPs may be preserved with high quality for 75 days at 25°C. After harvest, the application of chitosan and chitosan nanoparticles prevents decay, preserves fruit quality, and elongates shelf life. In contrast to the uncoated samples, which lost their overall acceptability scores mostly because of the ripening speed and bacterial infections, therefore displaying a low quality, the overall acceptability values were preserved in the fruits with nano coatings. Moreover, for farmers, we recommend the treatment of Valencia oranges with 0.8% nano-chitosan to obtain the best results for maintaining the marketable quality of Valencia oranges fruits under room temperature conditions.

## Data availability statement

The original contributions presented in the study are included in the article/**Supplementary Material**. Further inquiries can be directed to the corresponding authors.

## Author contributions

Conceptualization KA, MS, HA, MA, AH, SK; methodology, KA, MS, HA, MA, KH, SK, AE; software, AH, SK, MH, MG, AB, MI, KH, AE; validation; formal analysis, KA, MS, HA, MH, MA, AB, MI, KH, MA, AE; investigation, MH, MG, AB, MI, AY, AE resources, KA, MS, HA, MA, AH, SK, MH; data curation, MS, HA, MA, AH, SK, MH; writing—original draft preparation, MS, HA, MA, AH, SK, MH; writing—review and editing, KA, MS, HA, MA, AH, SK, MH, MG, AB, MI, KH, AE.; visualization, MG, AB, MI, KH, AE.; supervision, MH, MG, AB, MI, KH, AE; project administration, KA, MS, AE; funding acquisition, KA, MS, HA, MA, AH. All authors contributed to the article and approved the submitted version.

## Funding

This research work was funded by Princess Nourah bint Abdulrahman University Researchers Supporting Project number (PNURSP2022R188), Princess Nourah bint Abdulrahman University, Riyadh, Saudi Arabia.

## Acknowledgments

The authors thank Princess Nourah bint Abdulrahman University Researchers Supporting Project number (PNURSP2022R188), Princess Nourah bint Abdulrahman University, Riyadh, Saudi Arabia, for funding this research. We also thank the deanship of scientific research at Imam Mohammed Ibn Saud Islamic University, Riyadh, Saudi Arabia, for supporting publication of this research work. Our thanks also go to the Department of Horticulture, Faculty of Agriculture, Al-Azhar University, Cairo, Egypt.

## Conflict of interest

The authors declare that the research was conducted in the absence of any commercial or financial relationships that could be construed as a potential conflict of interest.

The reviewer AF declared a shared affiliation with the authors MS, HA, AH, SK, MH, MA, AB to the handling editor at the time of the review.

## Publisher’s note

All claims expressed in this article are solely those of the authors and do not necessarily represent those of their affiliated organizations, or those of the publisher, the editors and the reviewers. Any product that may be evaluated in this article, or claim that may be made by its manufacturer, is not guaranteed or endorsed by the publisher.
