# Supplementary figures and images for: Postharvest physiology and biochemistry of Valencia orange after coatings with chitosan nanoparticles as edible for green mold protection under room storage conditions
Source: Front Plant Sci. 2022 Nov 17;13:1034535. doi: 10.3389/fpls.2022.1034535 (PMC9745901; doi:10.3389/fpls.2022.1034535)

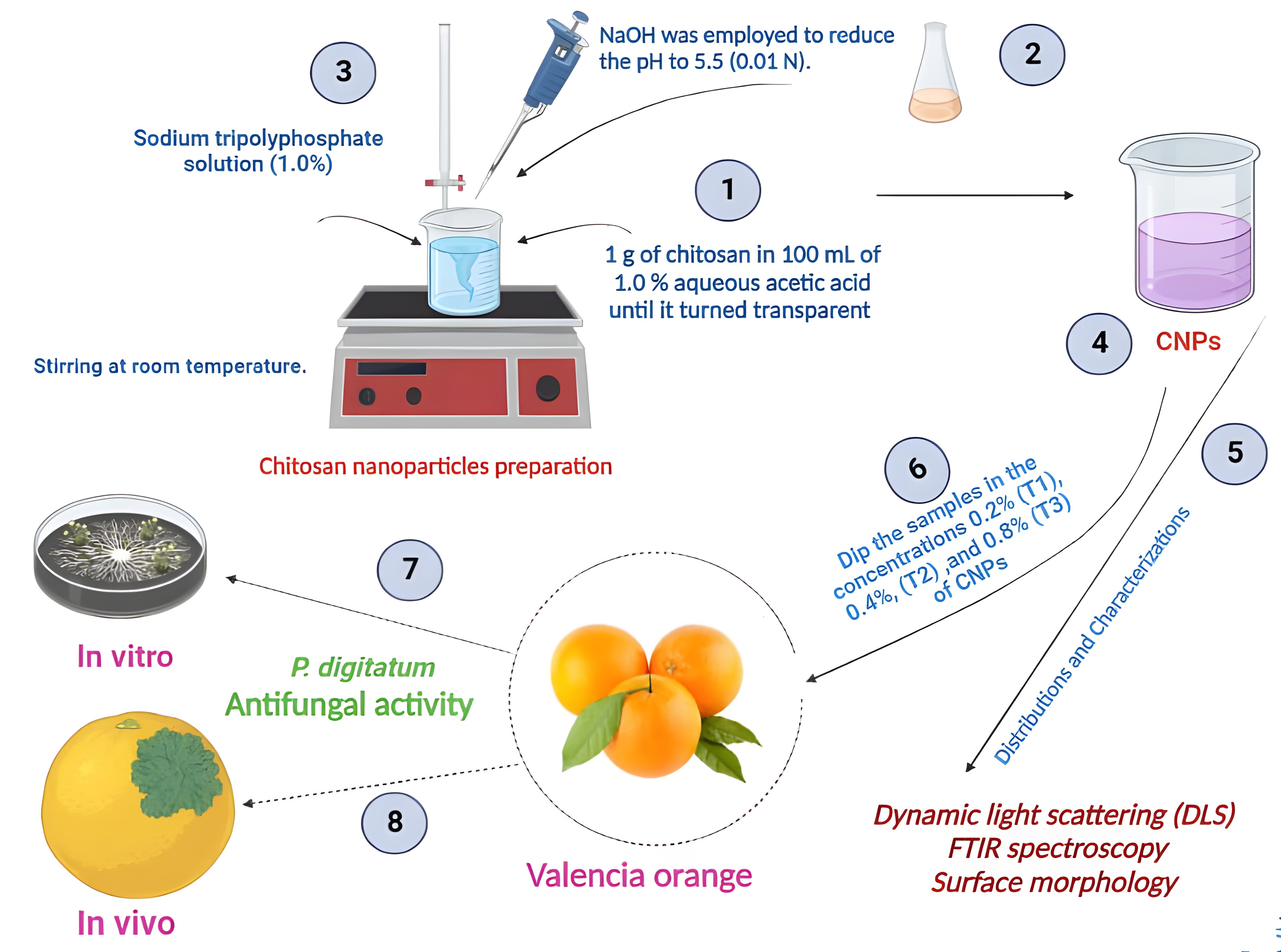

Supplement: Supplementary file 1 [file Image_1.jpeg]
